# Upward action promotes selective attention to negative words

**DOI:** 10.1016/j.heliyon.2021.e08394

**Published:** 2021-11-18

**Authors:** Yuki Nishiguchi, Shu Imaizumi, Yoshihiko Tanno

**Affiliations:** a Faculty of Human Sciences, Sophia University, 7-1 Kioi-cho, Chiyoda-ku, Tokyo, 102-8554, Japan; b Graduate School of Arts and Sciences, The University of Tokyo, 3-8-1 Komaba, Meguro-ku, Tokyo, 153-8902, Japan; c Institute for Education and Human Development, Ochanomizu University, 2-1-1 Otsuka, Bunkyo-ku, Tokyo, 112-8610, Japan

**Keywords:** Attention, Emotion, Embodied cognition, Motor action, Metaphor

## Abstract

Space-valence metaphors (e.g., bad is down) are embedded within cognitive and emotional processing (e.g., negative stimuli at a lower space capture visual attention more than those at an upper space). Previous studies have revealed that motor action to vertical direction affects the emotional valence rating of stimuli in a metaphor-congruent manner only when the action was introduced after the stimuli presentation. In the present study, we hypothesized that motor action before the stimuli presentation does not affect valence rating while it may affect visual selective attention. In Experiment 1 (participants: 28 university students; mean age = 19.50 years), we partially replicated the previous result with repeated ANOVA and *t*-tests; manual action introduced before the stimuli presentation does not affect the valence rating. Then, in Experiment 2 (participants: 28 university students; mean age = 19.57 years), we employed a modified version of the dot-probe task as a measure of visual selective attention to emotional stimuli, where participants’ vertical or horizontal manual action was introduced before the presentation of a pair of emotional words. The results of the *t*-tests revealed that an upward manual action promoting selective attention to *negative* words, which was incongruent with the space-valence metaphorical correspondence. These results suggest that even though manual action does not affect the evaluative process of emotional stimuli prospectively, upward manual action introduced before stimuli presentation can promote visual attention to the subsequent negative stimuli in a way that is incongruent with the space-valence metaphor.

## 1 Introduction

When we express our emotional experiences in daily life, we use spatial metaphors naturally. For example, when we want someone to feel better, we may say, “Cheer up.” Such space-valence metaphors do not merely appear in the form of slang or idioms; they exist in human cognition and emotions. In the early work of Wapner et al. (1957), participants bisected a luminous square with a black line before and after a midterm test. The results showed that the participants who succeeded in the midterm test tended to shift the bisecting line upward after the test, whereas those who failed shifted the line downward. In recent decades, such “metaphor-congruent” effects, which promote cognitive processes such as memory in a manner that is congruent with space-valence metaphors, have been continuously reported (Crawford and Cacioppo, 2002; Crawford et al., 2006; see also Lynott and Coventry, 2014). What is the mechanism underlying these effects of space-valence metaphors?

Lakoff and Johnson (1980, 1999) argued that abstract concepts are difficult to structure and define; thus, people use metaphors for more concrete and simple concepts that can be understood through daily experiences to help describe abstract concepts. For example, concepts like “prosperity” and “decline” are abstract and vague; thus, it may be difficult to understand them intuitively. As a result, we try to understand them by using metaphors like spatial metaphors (for example “prosperity is up, decline is down”), although such abstract concepts often require combinations of multiple metaphors to be understood thoroughly. Similarly, it could be assumed that people also use spatial metaphors to understand internal and external emotional signals, which are often vague and invisible.

Recently, researchers have demonstrated that emotions can affect visual attention in a metaphor-congruent manner. Meier and Robinson (2004) revealed that positive (or negative) evaluation of stimuli promoted information processing in the upper (or lower) area of the screen. This result suggests that the emotional valence of stimuli can affect spatial attention in a metaphor-congruent manner. Moreover, Meier and Robinson (2006) reported that stronger selective attention was allocated when neutral stimuli appeared in the lower half of the screen among participants with higher neuroticism, suggesting a “bad is down” metaphor on selective attention to visual stimuli, while there was no significant effect for “good is up”. Additionally, Zhang et al. (2014) reported that their participants felt happy when they gazed at the top of the screen and depressed when they gazed at the bottom, which shows that attention affects emotion in metaphor-congruent manner.

Another line of research reported evidence of interactions between body movement and emotion; for example, interactions between proprioception and emotion (e.g., Oosterwijk et al., 2009; Riskind and Gotay, 1982) and cutaneous sensation and emotional cognition (e.g., Fetterman et al., 2018). Some previous studies have observed the effect of motor action on emotional cognition (Förster & Strack, 1997, 1998; Maxwell and Davidson, 2007). Cognition that interacts with somatosensory and motor signals is called embodied cognition; more recently, studies have reported that vertical action corresponding to space-valence metaphors can modulate cognitive processing. Casasanto and Dijkstra (2010) reported that participants recalled their memories faster when movement and memory valence were metaphor-congruent, as upward movement promoted the recollection of positive memories while downward movement promoted the recollection of negative memories. Additionally, the findings presented by Sasaki et al. (2015) may serve as a supplement to those of Casasanto and Dijkstra (2010). In the study by Sasaki et al. (2015), emotional visual stimuli were presented on a touch screen, and subsequently, the participants manually moved a cursor upward, downward, or horizontally on the screen. When participants rated the emotional valence of the stimuli immediately after the upward (or downward) movement, the stimuli were found to be more positively (or negatively) rated. It seems reasonable that metaphor-congruent emotional processing is caused by vertical manual action, as suggested by evidence from embodied cognition.

Our previous study (Kato et al., 2018) replicated Sasaki et al. (2015) and included a condition where vertical manual action was introduced before the stimuli presentation. Subsequently, Kato et al. (2018) revealed that the valence rating was affected by the vertical manual action only retrospectively, not prospectively. Both Kato et al. (2018) and Sasaki et al. (2015), however, only examined the effect of metaphor-inductive action on valence rating. This absence of prospective effect can be specific to the valence-rating task. As previous studies including Casasanto and Dijkstra (2010) have paid attention to relatively higher-order cognition (memory or affective ratings), we should also explore lower-order cognitive features such as attention or perception to find prospective effects of manual action on emotional processing. The evidence of an interaction between action and attention has been studied for a long time (e.g., Gherri and Eimer, 2010; as a review, Smith and Schenk, 2012), and it is believed that metaphor-inductive action may affect visual attention. Thus, in the present study, we investigated the effect of metaphor-inductive action on attention to emotional stimuli.

In the present study, we introduced manual action before presenting emotional stimuli in order to investigate the effect of metaphor-inductive action. As introduced, spatial metaphor affects autobiographical memory or visual attention, thus we should expect a prospective effect of metaphor-inductive action on these cognitive aspects. According to these previous findings, the present study aimed at investigating the prospective effect of metaphor-inductive action on visual attention.

Though there is no previous report on the relationship between metaphor-inductive action and spatial attention, it is possible to expect similar results as shown in previous studies. For example, previous studies have observed that stimuli were processed faster when they were placed in a position congruent with a space-valence metaphor (e.g., Meier et al., 2007; Meier and Robinson, 2006). If metaphor-congruent effects promote the processing of emotional stimuli, detection of emotional stimuli may also be promoted (or may become the cause of faster reaction time [RT]) by vertical manual action. Thus, this study aimed to investigate the effects of vertical manual action on subsequent attention to negatively or positively valenced visual stimuli. The present study introduced manual action into a dot-probe task, which is an established task used to measure selective visual attention to emotional stimuli (MacLeod et al., 1986). Emotional words were presented after a manual action, and attentional bias (if any) towards the emotional verbal stimuli (words with emotional valence) was measured. At the same time, we attempted to replicate the result of Kato et al. (2018) to confirm that vertical manual action did not prospectively affect the valence rating of emotional stimuli at first, as Kato et al. (2018) used pictorial stimuli while the present study used emotional words as emotional verbal stimuli. Thus, in Experiment 1, we tested if vertical manual action affected the perceived valence of emotional stimuli in the present experiment setting. In Experiment 2, we employed a modified version of the dot-probe task and examined the effects of vertical manual action on the selective visual attention to the emotional stimuli used in Experiment 1. It can be expected that the manual action would not affect the valence rating of the subsequently presented emotional stimuli (Kato et al., 2018) in Experiment 1; however, attention would be affected by the orientation of manual action in metaphor-congruent ways, whereby upward movements promote attention to positive stimuli, and downward movements promote attention to negative stimuli, analogous to the findings of Casasanto and Dijkstra (2010) and Sasaki et al. (2015). Nevertheless, given the findings of Meier and Robinson (2006), we could expect the “bad is down” pattern to appear in attentional biases to emotional stimuli in Experiment 2.

## 2 Experiment 1

### 2.1 Methods

#### 2.1.1 Participants

A total of 28 university students (mean age = 19.50, *SD* = 0.84; 14 females) from introductory psychology classes at the University of Tokyo participated in this study. The sample size was based on a priori power analysis with G∗Power 3.1.9.3 (Faul et al., 2009). We assumed the large effect size (*d* = .80, according to the previous study; Kato et al., 2018) of a two-tailed one-sample *t*-test (alpha = .05, beta = .05), and found that a sample size above 23 was required for the present experimental design. Before the task began, participants answered the Japanese version (Okubo et al., 2014) of the Flinders Handedness Survey questions (Nicholls et al., 2013), which includes 10 items on hand usage in daily situations (scores ranged from -10 [left-handed] to 10 [right-handed]). As a result, all of the participants were right-handed except for one who was mixed-handed (*M* = 9.50, *SD* = 1.20). In the present study, we were interested in evaluating the spatial metaphor for upward and downward movement. The previous study by Casasanto (2009) showed that a spatial metaphor for vertical spatial direction (up/down) was not influenced by handedness. Moreover, the data showed that there was no difference between the left and right conditions in the present study (see 2.1.5 Analyses). Thus, the data of the mixed-handed participant were included in the present analyses. The participants also completed the Japanese version (Shima et al., 1985) of the Center for Epidemiological Studies Depression scale (CES-D; Radloff, 1977) because depression can affect judgment of emotional stimuli (Kohler et al., 2011). The CES-D is a self-report questionnaire that includes 20 items rated on a 4-point Likert scale, measuring the feelings of the participants during the past week (score range: 0 to 60). The CES-D score (*M* = 12.93, *SD* = 8.41) was comparable to the previous study on healthy Japanese community dwellers (*M* = 14.11, *SD* = 10.13; *N* = 1181; Takano et al., 2017) as the difference between these samples was small (Cohen's *d* = 0.13).

Each participant provided written informed consent before the experiment. The present study was conducted in accordance with the Declaration of Helsinki and was approved by the local ethical committee of the Graduate School of Arts and Sciences at the University of Tokyo (approval number 468).

#### 2.1.2 Materials

We selected 16 words each from the neutral, negative, and positive categories to use as emotional stimuli (see Appendix). Negative and neutral words were selected from familiarity-controlled word-sets collected from our previous study (Nishiguchi et al., 2015), originally from the word list of Matsumoto (2006). Positive words were selected from Matsumoto (2006), and their familiarity was matched to that negative and neutral words. An additional two neutral words were used in the practice trials.

#### 2.1.3 Apparatus

Participants were individually tested in a dark room in our laboratory. The experimental setup was mostly identical to the one used in Kato et al. (2018). Participants performed a rating task, sitting approximately 57 cm from a 24-inch liquid-crystal monitor with a refresh rate of 60 Hz (V242, Hewlett Packard, Palo Alto, USA) on the table. Their head positions were fixed using a chinrest. A joystick (Cyborg V.1 Flight Stick; Mad Catz, San Diego, USA) was positioned on a board erected perpendicularly to the table, as the bottom of the joystick was grounded on the board on the right side of the participant. Due to this setting, the direction of joystick manipulation and cursor movement are matched in the task explained in the following section. On the left side of the participant was a keyboard that was placed on a table; thus, each participant manipulated the joystick with their right hand and the numeric keypad with their left hand. Stimulus presentation and response collection were controlled by E-Prime 2.0 (Psychology Software Tools, Pittsburgh, USA) on a Windows 10 computer.

#### 2.1.4 Task

**Figure 1:**
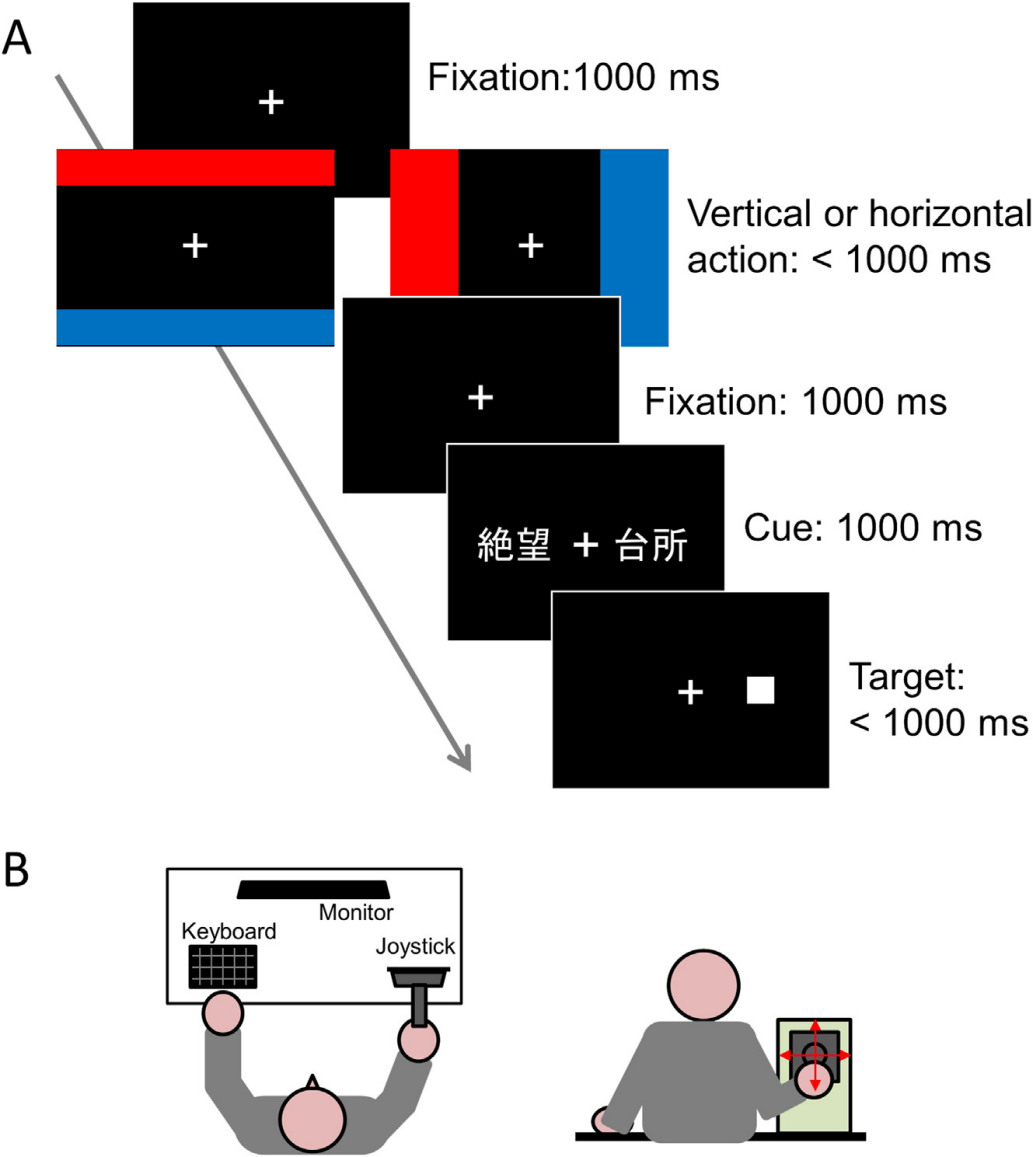
(A) Schematic procedure of the rating task in Experiment 1. (B) Schematic illustration of the present experiment setting.

The rating task (Figure 1) was mostly identical to that of Experiment 1 in Kato et al. (2018), without a retrospective condition. According to the orientation of the joystick manipulation, there were vertical and horizontal sessions. In the vertical session, the trial was started with the first fixation phase. A fixation cross (approximately 1.0° in width, 1.0° in height) appeared at the center of the screen for 1,000 ms, followed by the action phase. In the action phase, the fixation cross did not disappear, and red and blue bars (action cues, both approximately 51.8° in width, 8.1° in height) appeared at the top and bottom of the screen; at the same time, a cursor (a white cross, approximately 0.6 ° × 0.6 °), which could be manipulated with the joystick, appeared at the center of the screen. There was space between the bars at the top and bottom of the screen (51.8° in width, 14.5° in height). In half of the trials, a red bar appeared at the top of the screen, and in the other half, it appeared at the bottom of the screen. The colors of the bars, red and blue, were chosen to make the similar experiment setting as the previous studies (Kato et al., 2018; Sasaki et al., 2015). When the red and blue bars appeared, the participants were required to move the cursor to the red or blue bar within 1,000 ms. The destination of the cursor (the red or blue bar) was fixed for participants throughout the experiment, but was counterbalanced across the participants. In this manner, the participants moved the cursor in the upward direction for half of the trials and in the downward direction for the other half. If participants moved the cursor to the wrong bar or if the 1,000 ms mark was crossed, the trial was counted as an error and excluded from the analysis. After the participants either moved their cursors to the bar or when 1,000 ms elapsed, the bars and cursors were replaced by a 1,000 ms presentation of the second fixation phase. During this period, participants were required to return the joystick to the default (central) position to avoid any interference of the vision and movement of their arm on the subsequent stimulus presentation. Notably, action and its sensory outcome can be temporally bound to one another, even with temporal intervals longer than 1,000 ms (Humphreys and Buehner, 2009). Next, the cue presentation phase started with one of the emotional words (approximately 2.0 ° × 1.0 °) appearing at the center of the screen for 1,000 ms. Then, the participants rated the emotional valence of the word on a 7-point Likert scale ranging from -3 (strongly negative) to 3 (strongly positive) by pressing a numeric key horizontally aligned on the keyboard (1–7 keys; i.e., the 1 key for “-3” and the 7 for “3”) with no time pressure. This took place in one experimental block. There were eight trials each for all combinations of action orientations (upward and downward) and stimulus valences (negative, neutral, and positive), resulting in 48 trials. There was also the horizontal session, in which only the locations of the action cues were different from those in the trials of the vertical session. The red and blue bars appeared to the left and right of the central fixation. Both bars were approximately 18.7° in width and 32.4° in height, matching the width of the space between the two bars (i.e., they required an amount of arm displacement), and were approximately the same as the vertical session (14.5°). The number of trials and block design was the same as in the vertical session. The order of the sessions was counterbalanced across participants.

In the present task, the joystick manipulation before the stimuli presentation required both arm flexion and contraction because participants had to move the cursor by manipulating the joystick, and then move it back to the default position. Some previous studies had tasks which required both arm flexion and contraction in one trial and successfully found a metaphor-congruent effect (Casasanto and Dijkstra, 2010; Kato et al., 2018). Thus, we believe that this requirement did not alter the results.

#### 2.1.5 Analyses

First, we averaged the ratings of the trials for each condition and participant and confirmed that there was no significant difference in the rating scores between the left and right conditions (*p*-values are Bonferroni-corrected for three *t*-tests; negative stimuli, *t* (27) = 0.07, *p* = 1.000, *d* = 0.02; neutral stimuli, *t* (27) = -1.02, *p* = .946, *d* = 0.17; positive stimuli, *t* (27) = -0.83, *p* = 1.000, *d* = 0.14) thus, we averaged the scores of left and right conditions and treated them as a horizontal condition. A two-way repeated measure (stimuli valence and orientation) ANOVA was conducted on the valence ratings.

Following this, we calculated the changes in valence rating to examine the change caused by upward and downward joystick manipulation, compared to the horizontal condition. The changes in valence ratings were calculated by subtracting the average rating score in the horizontal condition from that in the upward and downward conditions for negative, positive, and neutral words. Then, for the main purpose of Experiment 1, we conducted *t*-tests to examine whether upward and downward manual action changed valence rating, following the analyses procedure of Kato et al. (2018). Finally, a correlation analysis was conducted to examine the effect of depressive symptoms on the change of valence ratings.

### 2.2 Results

We conducted a two-way repeated measure ANOVA on the valence ratings, with stimuli valence (negative, positive, neutral) and orientation (upward, downward, horizontal) as within-participants factor. There was a significant main effect of valence (*F* (2, 54) = 1022.66, *p* < .001, *η*2 ​p = .974) without interaction from orientation (Greenhouse–Geisser corrected *F* (1.30, 35.19) = 0.25, *p* = .683, *η*2 ​p = .009). There was no main effect of orientation (Greenhouse–Geisser corrected *F* (2.66, 71.78) = 2.63, *p* = .063, *η*2 ​p = .089).

**Figure 2:**
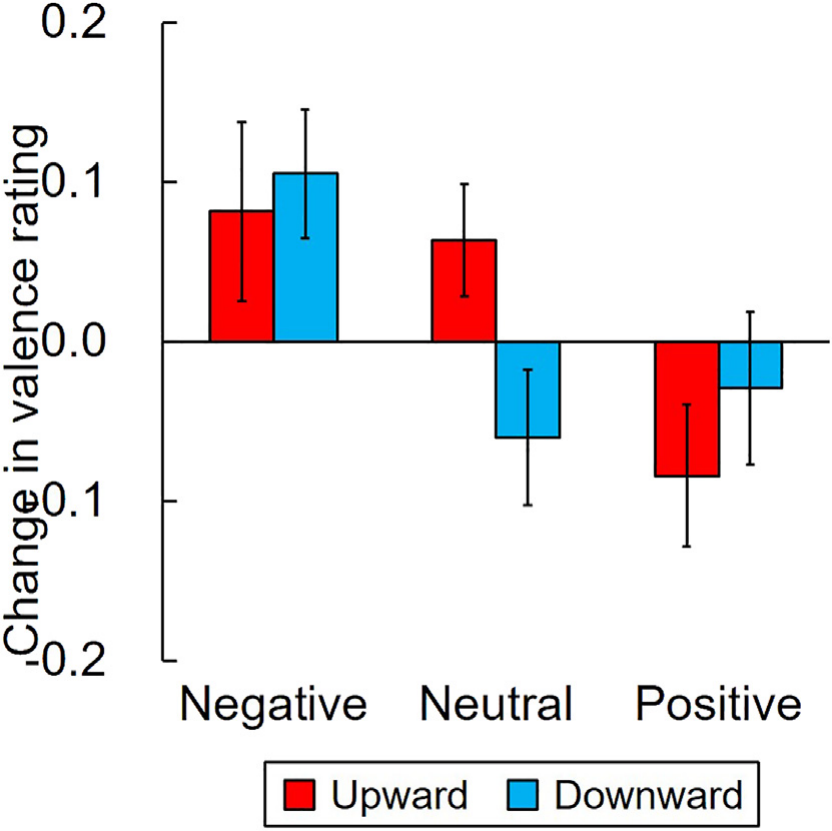
Changes in valence ratings for negative, neutral, and positive stimuli caused by vertical manual action in comparison to horizontal action in Experiment 1. Error bars represent the standard error of the mean.

Then, for the primary purpose of the present experiment, we calculated the changes in valence rating in the upward and downward conditions for negative, positive, and neutral words (Figure 2). We examined the difference between the changes in valence ratings and zero, with a one-sample *t*-test with a Bonferroni correction (*p*-values were corrected for six *t*-tests). However, no significant differences were found, while negative stimuli in a downward condition showed a modest effect size (negative stimuli in a downward condition, *t* (27) = 2.61, *p* = .090, *d* = 0.49 and in an upward condition, *t* (27) = 1.45, *p* = .948, *d* = 0.27; neutral stimuli in an upward condition, *t* (27) = 1.79, *p* = .504, *d* = 0.34 and in a downward condition, *t* (27) = -1.42, *p* = .996, *d* = 0.27; positive stimuli in an upward condition, *t* (27) = -1.88, *p* = .426, *d* = 0.35 and in a downward condition, *t* (27) = -0.61, *p* = 1.000, *d* = 0.12), suggesting that no change was caused by manual action on the emotional valence perception of the stimuli.

The correlation between the CES-D score and the changes in valence rating was also tested. No significant correlation was found, however. Specifically, for negative stimuli in an upward condition we found *r* (26) = -.080, *p* = .686; for a downward condition, *r* (26) = .231, *p* = .237; for neutral stimuli in an upward condition, *r* (26) = .195, *p* = .320 and in a downward condition, *r* (26) = -.273, *p* = .160; for positive stimuli in an upward condition, *r* (26) = -.234, *p* = .160 and in a downward condition, *r* (26) = .241, *p* = .216.

## 3 Experiment 2

### 3.1 Methods

#### 3.1.1 Participants

A total of 28 university students (mean age = 19.57, *SD* = 1.45; 17 females—none of whom participated in Experiment 1) from the classes of an introductory psychology course at the University of Tokyo participated in this segment of the study. The sample size was determined in the same way as Experiment 1. In accordance with the Flinders Handedness Survey (Okubo et al., 2014), three participants were found to be mixed-handed, but their data were included in the present analyses for the same reason as Experiment 1. The others were right-handed. The average score of all participants was 8.48 (*SD* = 3.91). The participants also completed the CES-D (Radloff, 1977), because depression can affect attentional bias to emotional stimuli (Peckham et al., 2010). The CES-D score of the present participants (*M* = 11.39, *SD* = 7.00) was comparable to healthy Japanese community dwellers (Takano et al., 2017) as the difference between these samples was relatively small (*d* = 0.31). All participants had normal or corrected-to-normal visual acuity.

#### 3.1.2 Materials

A total of 48 emotional words identical to those in Experiment 1 were used.

#### 3.1.3 Tasks

**Figure 3:**
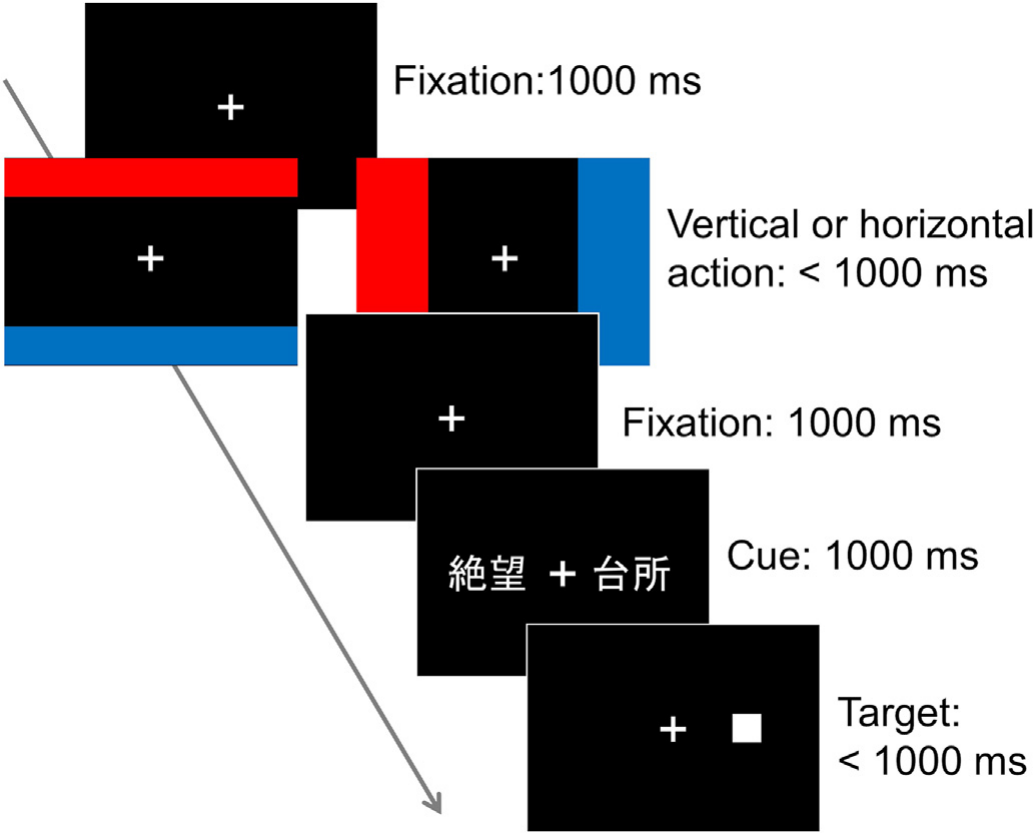
Schematic procedure of the modified dot-probe task in Experiment 2.

In the modified dot-probe task, motor action through joystick manipulation was introduced (Figure 3; MacLeod et al., 1986). The first fixation phase, action phase, and the second action phase were identical to those of the rating task in Experiment 1. After the second fixation phase, the cue presentation phase started with a pair of words appearing to the left and right of the fixation cross for 1,000 ms. These words were approximately 3.0° from the center of the fixation cross (measured from the center of the words). The word pairs were always composed of an emotional word (negative or positive) and a neutral word, written in two-lettered kanji, subtending approximately 2.0° in width and 1.0° in height. Immediately after the word pair disappeared, a white square target (0.6 ° × 0.6 °) was presented at the location where the word had been present. The participants were required to report the location of the target (left or right) by pressing a key (“1” key on the numeric keypad for “left” and “3” for “right”) within 1,000 ms. When a response was given or 1,000 ms had passed without a response, the trial was considered an error trial and the next trial was immediately started.

In the modified dot-probe task, there were congruent trials and incongruent trials; the target appeared in the same place as the emotional words during the congruent trials, but in the opposite spot during the incongruent trials. As both negative and positive words were used as emotional words, there were four types of trials: congruent-negative, incongruent-negative, congruent-positive, and incongruent-positive trials. Participants completed a block of practice, followed by two experimental blocks. In each experimental block, there were eight trials each for combinations of action orientations (upward and downward) and trial types (congruent-negative, incongruent-negative, congruent-positive, and incongruent-positive), resulting in 64 trials in total. The trial order was randomized within participants. A break was provided between blocks.

As in the rating task, there were vertical and horizontal sessions. The number of trials and block design was the same as in the vertical session. The order of the sessions was counterbalanced across participants.

#### 3.1.4 Data cleaning and analyses

Before the analyses, error trials and trials with extremely short or long RTs (mean ± 2*SD*) were excluded (1.7% of the total). We averaged the RTs of the trials for each condition and participant, then calculated changes in attentional bias to examine whether attention to emotional stimuli was changed by upward or downward movement. Then, we calculated the bias index (Koster et al., 2004; Mogg et al., 2000). The negative bias index was calculated by subtracting the congruent-negative trial RTs from the incongruent RTs for each upward, downward, left, and right condition. Similarly, the positive bias index was also calculated. We found that there was no significant difference between the negative and positive bias indices between the left and right conditions: *p*-values Bonferroni-corrected for two *t*-tests; negative bias index, *t* (27) = -0.39, *p* = 1.000, *d* = 0.11; positive bias index, *t* (27) = 0.51, *p* = 1.000, *d* = 0.16. Thus, the average of the bias indices for the left and right conditions was used as the bias index for the *horizontal* condition. A two-way repeated measure ANOVA was conducted on the bias index. Then, as in Experiment 1, we conducted *t*-tests with a Bonferroni correction for the main purpose of examining whether upward and downward manual action changed attention to emotional stimuli following the procedure of Kato et al. (2018). Finally, a correlation analysis was conducted to examine the effect of depressive symptoms on the change of valence ratings.

The changes in the bias indices due to vertical action were calculated by subtracting the bias index for the horizontal condition from that of the upward and downward conditions (Sasaki et al., 2015; Kato et al., 2018). For example, the change in the bias index for the positive stimuli in the upward condition was calculated by subtracting the positive bias index in the horizontal condition from the positive bias index for the upward condition. Accordingly, the changes in the bias index for the negative and positive stimuli in the upward and downward conditions were calculated.

### 3.2 Results

A repeated measures ANOVA with *valence* (negative, positive) and *orientation* (upward, downward, horizontal) as within-participants factors was performed on the change in bias index. The main effects of valence (*F* (1, 27) = 0.04, *p* = .835, *η*2 ​p = .002) and orientation (*F* (2, 54) = 2.98, *p* = .061, *η*2 ​p = .099) were not significant. The interaction between valence and orientation was also not significant (Greenhouse–Geisser corrected, *F* (1.53, 41.18) = 1.13, *p* = .318, *η*2 ​p = .040).

**Figure 4:**
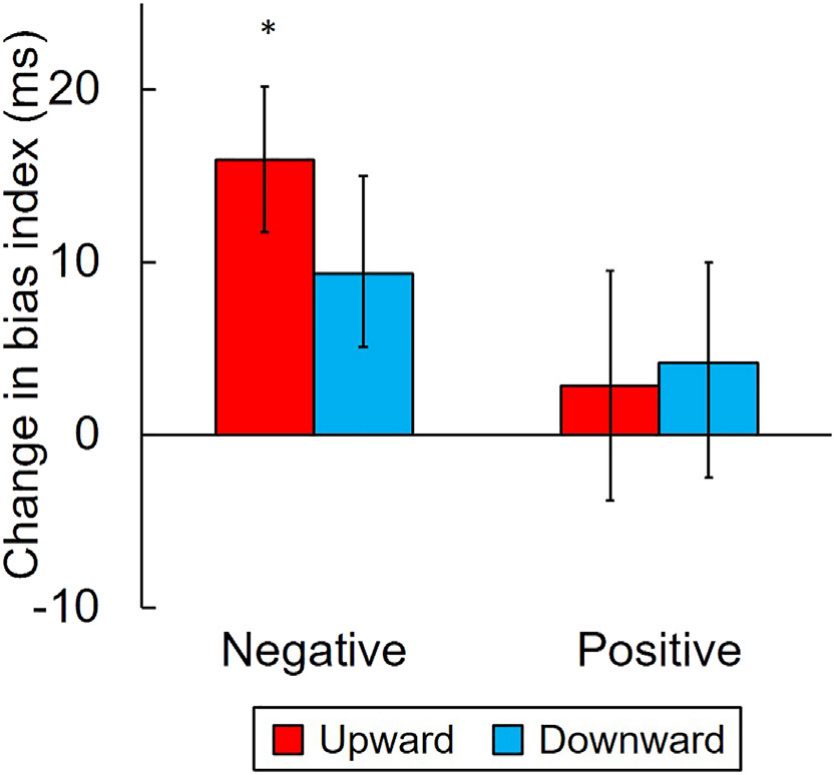
Changes in bias index for negative and positive stimuli caused by vertical manual action in comparison to horizontal action in Experiment 2. Asterisk represents a significant difference between the change in bias index and zero (∗ p < .05, two-tailed, Bonferroni-corrected). Error bars represent the standard error of the mean.

For the primary purpose of Experiment 2, we investigated whether vertical action caused any changes in attentional bias by conducting a one-sample two-tailed *t*-test against zero of the changes in bias indices (Figure 4) with a Bonferroni correction (corrected for four *t-*tests). The change in the bias index for negative stimuli in the upward condition was significantly higher than zero (*t* (27) = 2.80, *p* = .036, *d* = 0.53), which indicates that upward movement increased attention to negative stimuli. No other changes were significantly different from zero (negative bias in downward condition, *t* (27) = 1.59, *p* = .496, *d* = 0.30; positive bias in the upward condition, *t* (27) = 0.68, *p* = 1.000, *d* = 0.13; positive bias in the downward condition, *t* (27) = 0.62, *p* = 1.000, *d* = 0.12; *p*-values Bonferroni-corrected).

The correlation between the CES-D score and the changes in bias index was also tested; however, no significant correlation was found. Our results showed negative bias in the upward condition *r* (26) = -.098, *p* = .621 and in the downward condition, *r* (26) = .210, *p* = .284; positive bias in the upward condition, *r* (26) = -.057, *p* = .772 and in the downward condition, *r* (26) = .096, *p* = .626. This suggests that depressive symptoms did not affect the changes in attentional bias caused by manual action.

## 4 Discussion

In the present study, we investigated the effects of vertical and horizontal manual movements on ratings of the emotional valences of the stimuli and attention to subsequently presented emotional stimuli. Experiment 1 showed no effect of manual movements introduced before the stimuli presentation on emotional valence rating. Experiment 2 showed that manual action before the stimuli presentation only influenced attention to negative stimuli when upward action was introduced, while downward action did not affect attention. The present study revealed that metaphor-inductive manual action prospectively affects attention to emotional verbal stimuli, as this was the main investigative focus. The effects of manual action, however, were found to be incongruent with space-valence metaphors.

### 4.1 Relationship between metaphor-inductive vertical action and emotional attention

The results of Experiment 2, which showed that upward action promoted attention to negative stimuli, demonstrated a connection between metaphor-inductive manual action and attention. As discussed above, vertical manual action has been known to affect valence rating (Kato et al., 2018; Sasaki et al., 2015) or autobiographical memory (Casasanto and Dijkstra, 2010). It is assumed that the metaphorical association between space and valence was activated by manual action, leading to an increased occurrence of metaphor-congruent information processing in these studies. As Experiment 1 did not show any effects of the action on the emotional ratings of the stimuli, perceptions of the emotional valence of the stimuli were not affected prospectively by manual actions. Thus, the metaphor-inductive manual action prospectively affected only attention. However, the effect is independent of the change in valence perception, which was only observed when action is introduced after the stimuli presentation (Kato et al., 2018; Sasaki et al., 2015).

Then, how did action cause the change in selective attention to emotional stimuli? For a possible explanation, it has been reported that mood or motivational state can affect attentional bias (Mogg and Bradley, 1998). The change in attention was possibly caused by changes in mood or motivational state evoked by metaphor-inductive action. Action changes a mood or motivational state, according to previous studies on embodied cognition (Niedenthal, 2007; but see Wagenmakers et al., 2016, for a controversy), although it is unclear as to whether a minimal action (like joystick manipulation) is enough to change the internal mood state. Additionally, it is also possible that the attentional bias changed without any change in the internal mood state. The metaphorical effect of action, which is assumed to be caused by the activation of a metaphorical association between valence and space, may not necessarily co-occur with a change in internal mood state, as seen in a priming effect (Silvia et al., 2006; Soldat and Sinclair, 2001). Nevertheless, it would be interesting to compare the mood between before and after a task to examine the total effect of the task on the mood state of participants.

### 4.2 Metaphor *incongruent* effect of vertical action

In some ways, the present results were not congruent with previous results. The largest incongruence with the previous studies was the finding that upward movement promoted attention to negative stimuli but not to positive stimuli, which does not match the “good is up” metaphor. The present study employed different methods from those in other studies, and there was no previous report of metaphor-incongruent effect resulting from metaphor-inductive action. However, a previous study reported that negatively valenced words were recalled better when they were placed at the upper space of the screen (Crawford et al., 2014); this kind of incongruency is possibly observed in the space-valence metaphor (while Crawford et al. (2014) themselves did not find a significant effect of manual action).

We may be able to explain this metaphor-incongruent effect according to another line of research; for example, the mood-incongruent effect. Previous studies have found that people sometimes seek information with emotional valence that is different from their mood state (Forgas, 1995), although people normally process mood-congruent information better (Bower, 1981). This effect is called the mood-incongruent effect. The mood-incongruent effect arises to moderate ones’ internal mood state. It is assumed that mood-incongruent information is sought when we are motivated to moderate the current mood state or when some time has passed from the arising of a mood (Forgas and Ciarrochi, 2002). Though these conditions do not seem to apply to the present experimental setting, incongruent information processing evoked by the valence-space metaphor may occur within some different conditions.

Joystick manipulation might have otherwise stimulated the unexpected effect of embodied cognition, which disturbed the effect of the space-valence metaphor. Cacioppo, Priester, and Berntson (1993) observed that arm flexion stimulated a positive evaluation of presented stimuli, while arm extension stimulated a negative evaluation. Cacioppo et al. (1993) assumed that arm flexion is related to approaching behavior (e.g., pulling an object), while the extension is related to avoidant behavior (e.g., pushing an object away). In the present experiment, upward manual action included arm extension, which may lead to negatively biased information processing. It should be noted that the participants were required to return the joystick to its default position after every trial in the action phase, which means that the participants always had to make downward manual actions after an upward action, to return the joystick to the default position, and vice versa. In other words, both arm extension and flexion were present in every trial in the vertical session. Thus, the mere physical movement does not seem to explain the present results, as reported by Casasanto and Dijkstra (2010). According to Eder and Rothermund (2008), if participants labeled their movement as “pushing joystick away,” negative information processing may be promoted, even when the joystick and cursor were moved upward. Referring to Casasanto and Dijkstra and Sasaki et al. (2015), we did not instruct participants to “move cursor upward/downward,” but instead instructed them to “move the cursor to the red/blue bar” to observe the effect of vertical manual action without semantically labeling the vertical space. Nevertheless, it is possible that the participants voluntarily labeled their actions, which requires a somewhat different motor action from that in the marble lifting (Casasanto and Dijkstra, 2010) and touch panel manipulation (Sasaki et al., 2015) used in previous studies. Overall, we cannot specify the mechanism underlying the present results as there are few previous shreds of evidence on the relationship between action and attention. It is hoped that future studies will systematically investigate the relationship between the present results and previous studies, like Casasanto and Dijkstra or Sasaki et al., to further enhance our understanding. More specifically, the effect of arm flexion and contraction can be examined if the joystick returned to the default position automatically after the participants moved the cursor and released the joystick in the future experiment.

Another incongruency with previous studies was that action only increased attention to negative stimuli and had no apparent effect on attention to positive stimuli. However, this result is not surprising, as previous studies suggest that a metaphorical association between negative valence and lower position is stronger than the association between positive valence and an upper position (Crawford and Cacioppo, 2002; Meier and Robinson, 2006). Particularly, Meier and Robinson (2006) reported only the “bad is down” effect in visual selective attention. Thus, the negativity bias in the space-valence metaphor may be considered as one cause of the absence of the action effect on attention to positive stimuli. Additionally, in a line of previous attentional bias studies, it is often reported that attentional bias to positive stimuli is rarely observed, as compared to an attentional bias to negative stimuli (Fox et al., 2002). Detecting negative information is more biologically and evolutionally important for survival than positive information (Crawford and Cacioppo, 2002; Shweder et al., 2008; Tovote et al., 2015). Thus, attentional bias to negative stimuli is often more prominent than attentional bias to positive stimuli.

### 4.3 Limitations

There were some limitations in the present methods. The first limitation is that the present study could not examine the time course of the metaphorical effects of an action. In the present study, the action was always inserted one second before the presentation of emotional stimuli. As Kato et al. (2018) and Sasaki et al. (2015) found, not only the order of action and stimuli presentation, but also the temporal proximity between action and stimuli, can affect emotional information processing. An action may also be introduced during the stimuli presentation, as reported by Casasanto and Dijkstra (2010). A time-course effect on metaphor-inductive action should be systematically investigated in future studies.

Second, the apparatus in the present study might not have been optimized. We referred to previous studies (Kato et al., 2018; Sasaki et al., 2016) to develop the present experimental settings. However, it is unclear whether the amount or intensity of action was appropriate. For example, there was asymmetry in the difficulty of the upward and downward manipulations in the present study due to gravity. This may have caused a somewhat negative nuance or influence on upward manual action. Thus, future studies should carefully investigate the potential effects of various motor properties (e.g., loads, speeds, and directions) on emotional and metaphorical processing.

Additionally, the present study used a dot-probe task, which is a task to measure visual selective attention. There are many other kinds of visual attention tasks as well. For example, Posner's cueing paradigm (Posner, 1980) can measure attentional engagement and disengagement separately. Visual search paradigms, or the monitoring of eye-gaze, are also useful ways to measure attentional bias. Moreover, the present results are limited to visual attention. However, the metaphorical effect is possibly observed in auditory attention, as attention to verbal stimuli was affected by metaphor-inductive action. In these ways, the effect of the space-valence metaphor in various aspects of attention can be investigated in future studies.

### 4.4 Conclusions

The present study investigated the effects of motor action introduced before the emotional stimuli presentation and space-valence metaphors on the valence rating of emotional stimuli and attention to emotional information. The results showed that valence rating was not affected, while selective attention to emotional words was affected by manual action. However, only the upward action increased attention to negative stimuli, which was not congruent with space-valence metaphors. It is hoped that the effects of the experimental setting, including the time course effects of the action and stimuli presentation, on attention will be investigated in detail in future studies. Future studies should examine the effect of metaphor-inductive action on emotional processing and expand these findings into the field of prospective memory. In the present study, it was suggested that vertical manual action prospectively affects attention to emotional stimuli; however, the effect was metaphor-incongruent. On the other hand, the previous studies have shown that manual action retrospectively affects emotional information processing in a metaphor-congruent manner (Kato et al., 2018; Sasaki et al., 2015). Taken together, the prospective effect of metaphor-inductive action can be inverted from a retrospective one, and we can expect that the metaphor-incongruent effect of action will affect prospective memory (or, there may be no effect as Kato et al. showed). In this way, future research can expand present findings into other fields of cognitive psychology.

These experiments suggested that attentional change by manual action may be independent of change in valence perception. Previous studies have discussed the association between action and attention (e.g., Smith and Schenk, 2012), and additionally, the present study suggested that spatial metaphor may be associated with this relationship. If the knowledge on the relationship between metaphor-inductive action and attention is accumulated in the future, it can be practically applied to various fields. For example, the present results suggest that if we want people to attend negative information (e.g., precautionary statements), we can introduce upward action before the presentation of them (e.g., people have to raise a lever to get tickets for a vending machine). We can apply the present findings to work in clinical psychology, as metaphor-inductive motor action may help patients with depression or anxiety to overlook or ignore negative information. For example, inhibition of vertical manual action (especially upward action) can decrease attention to negative stimuli. Future research should explore such practical applications of metaphor-inductive action.

## Declarations

### Author contribution statement

Yuki Nishiguchi: Conceived and designed the experiments; Performed the experiments; Analyzed and interpreted the data; Contributed reagents, materials, analysis tools or data; Wrote the paper.

Shu Imaizumi: Conceived and designed the experiments; Analyzed and interpreted the data; Contributed reagents, materials, analysis tools or data; Wrote the paper.

Yoshihiko Tanno: Contributed reagents, materials, analysis tools or data; Wrote the paper.

### Funding statement

This work was supported by Grants-in-Aid for JSPS Research Fellow (16J00411, 17J02455) and Young Scientists (B) (17K12701) from the 10.13039/501100001691Japan Society for the Promotion of Science.

### Data availability statement

The authors do not have permission to share data.

### Declaration of interests statement

The authors declare no conflict of interest.

### Additional information

No additional information is available for this paper.

## Figures and Tables

**Table:** 

Practice	Negative	Neutral	Positive
角度 (angle)	混雑 (crowded)	任期 (tenure)	優勝 (win the championship)
石油 (petroleum)	迷惑 (nuisance)	呑気 (carefree)	陽気 (merry)
	憂鬱 (depression)	部品 (parts)	裕福 (wealthy)
	後悔 (regret)	印象 (impression)	親友 (close friend)
	死亡 (death)	勝敗 (results)	誠実 (sincere)
	拒絶 (refusal)	逆算 (inversion)	敬愛 (adoration)
	無力 (helplessness)	座席 (seat)	感銘 (deeply impressed)
	中毒 (addiction)	染料 (dye)	爽快 (exhilarating)
	全滅 (annihilation)	体調 (condition)	勝利 (victory)
	気絶 (faint)	脈拍 (pulsation)	安心 (relief)
	敗者 (loser)	朗読 (reading aloud)	信頼 (trust)
	敵意 (hostility)	浴槽 (bathtub)	大吉 (great fortune)
	危害 (harm)	呼吸 (breath)	優良 (excellent)
	苦手 (dislike)	近況 (recent state)	安全 (safety)
	卑劣 (mean)	発言 (remark)	好感 (likable)
	陰気 (gloomy)	上司 (boss)	善意 (goodwill)
